# Role of Cattaneo–Christov heat flux in an MHD Micropolar dusty nanofluid flow with zero mass flux condition

**DOI:** 10.1038/s41598-021-98988-5

**Published:** 2021-09-30

**Authors:** Muhammad Ramzan, Hina Gul, Dumitru Baleanu, Kottakkaran Sooppy Nisar, M. Y. Malik

**Affiliations:** 1 Department of Computer Science, Bahria University, Islamabad, 44000 Pakistan; 2 Department of Mathematics, Cankaya University, 06790 Ankara, Turkey; 3 Institute of Space Sciences, 077125 Magurele-Bucharest, Romania; 4 Department of Medical Research, China Medical University Hospital, China Medical University, Taichung, 40447 Taiwan; 5 Department of Mathematics, College of Arts and Sciences, Prince Sattam Bin Abdulaziz University, Wadi Aldawaser, 11991 Saudi Arabia; 6 Department of Mathematics, College of Sciences, King Khalid University, Abha, 61413 Saudi Arabia

**Keywords:** Mechanical engineering, Software

## Abstract

This investigation aims to look at the thermal conductivity of dusty Micropolar nanoliquid with MHD and Cattaneo–Christov heat flux flow over an elongated sheet. The novelty of the envisioned mathematical model is augmented with the added impacts of the heat source/sink, chemical reaction with slip, convective heat, and zero mass flux boundary conditions. The salient feature of the existing problem is to discuss the whole scenario with liquid and dust phases. The graphical depiction is attained for arising pertinent parameters by using bvp4c a built-in MATLAB function. It is noticed that the thermal profile and velocity field increases for greater values of liquid particle interaction parameter in the case of the dust phase. An escalation in the thermal profile of both liquid and dust phases is noticed for the magnetic parameter. The rate of mass transfer amplifies for large estimates of the Schmidt number. The thickness of the boundary layer and the fluid velocity are decreased as the velocity slip parameter is augmented. In both dust and liquid phases, the thermal boundary layer thickness is lessened for growing estimates of thermal relaxation time. The attained results are verified when compared with a published result.

## Introduction

Nanofluid is a combination of nanometer-sized particles and a base fluid that helps to improve the heat capacity of the solution. The addition of millimeter or micrometer small particles (dust particles) to base fluids improves thermal conductivity and is referred to as Dusty fluid. The influence of Micropolar dust particles with MHD in a non-Darcy porous system is observed by Hady et al.^1^. It is witnessed in this analysis that the velocity magnitude for both dust and fluid phases boosts for variable concentration. It is also noticed that the temperature upsurges for the Darcy number and convective parameter. Begum et al.^2^ analyzed numerically the Dusty nanoliquid of gyrotactic microorganisms along a vertical isothermal surface. Nabwey and Mahdy^3^ investigated dusty particles with a nonlinear temperature of Micropolar natural convection nanoliquid flow past a permeable cone. It is discovered from the results that increasing the suction variable boosts the local Nusselt number. Nabwey and Mahdy^4^ in another study examined unsteady non-Newtonian hybrid nanoliquid flow filled with Fe_3_O_4_–Ag dust nanoparticles over a stretched surface under the influence of MHD free convection with surface temperature and a prescribed heat flux of boundary conditions. The numerical solution of the problem is acquired using a Finite Difference Method. Some recent studies highlighting nanoliquid flow may be found in^5–23^ and many therein.

Owing to enormous applications in nanofluid mechanics, researchers are working on the heat transfer mechanism in the form of a wave instead diffusion process^24–26^. It is a understood phenomenon that the transfer of heat occurs owing to temperature differences amongst two different objects or within the components of the same system. The basic heat conduction law coined by Fourier^27^ has been a yardstick for decades to gauge the heat transfer characteristics. Later, it was noticed with concern that this model ends up with a parabolic energy equation that experiences a disturbance at an initial stage that lasts throughout the process. This drawback in the Fourier model is signified as a “paradox of heat conduction”. This shortcoming is addressed by Cattaneo^28^ by introducing the relaxation term in the Fourier model. Later, Christov^29^ established the relation suggested by Cattaneo through frame-indifferent change with the Oldroyd upper-convected derivative. Such association is labeled as Cattaneo–Christov (CC) flux model. Kumar et al.^30^ researched the characteristics of Dusty fluid of suspended hybrid nanoparticles flows in two phases over an extended cylinder with a CC flux model. For numerical results, the fourth fifth Runge–Kutta–Fehlberg order system was used, as well as the shooting methodology. It is noticed that the thermal profile and thickness of the thermal boundary layer are higher for the relaxation time parameter due to the melting effect. Ramzan et al.^31^ analyzed the Williamson fluid flow numerically with the CC flux model and magnetohydrodynamic effect with heterogeneous reactions near a stagnation point. It is noted that the Williamson fluid parameter has an opposing effect on temperature and velocity profiles. Prasad et al.^32^ conducted an analytical study of Williamson nanofluid flow with the Cattaneo–Christov theory using variable thickness. Heat transfer examination of non-Newtonian nanoliquid flow with convective boundary conditions and CC flux model over an oscillatory surface is assessed analytically by Ullah et al.^33^. It is examined that the liquid velocity is suppressed for Hartmann and Deborah numbers.

The aforementioned studies disclose that plenty of explorations may be quoted on the subject of nanofluid flows. Nevertheless, fewer researches are pondered in the literature that signify the nanofluid flow with dust particles amalgamation. But no study is discussed so far in the literature that pondered the Cattaneo–Christov heat flux on an MHD Micropolar dusty nanofluid flow over a stretched surface with slip, convective heat, and zero mass flux conditions. The originality of the modeled problem is augmented with the additional impacts of the chemical reaction and heat source/sink. Thus, the association of dust particles, Micropolar nanofluid, and slip, convective, and zero mass flux condition boundary conditions is supposed to present a remarkable problem in liquid dynamics based on these physical assumptions. To portray a clear picture of the uniqueness of the present analysis Table 1 is erected by comparing it with the associated published works.

**Table 1:** Assessment of the present work with the close related published works.

	Dusty fluid	Nanofluid flow	Velocity slip	Zero mass flux	Cattaneo Christov heat flux	Micropolar fluid	Chemical reaction	Convective boundary
Hady et al.^1^	Yes	No	No	No	No	Yes	No	Yes
Gireesha et al.^35^	Yes	No	No	No	Yes	No	No	No
Begum et al.^2^	Yes	Yes	No	No	No	No	No	No
Souayeh et al.^36^	Yes	Yes	Yes	No	No	No	No	No
Anuar et al.^37^	Yes	Yes	Yes	No	No	No	No	No
Present work	Yes	Yes	Yes	Yes	Yes	Yes	Yes	Yes

The prime objective of the presented model is to answer the subsequent answers:

1. How fluid velocity and temperature are affected by fluid-particle interaction effects?
2. How dust and fluid phases for velocity and temperature profiles are influenced by the magnetic parameter?
3. What is the association of the thermal relaxation parameter with the fluid velocity and the temperature in case of both phases?
4. How fluid velocity is influenced by the slip parameter?
5. What is the impact of the chemical reaction on the rate of the mass transfer?
6. How fluid temperature is affected by the heat source/sink for both liquid and dust phases?

## Mathematical model

**Figure 1:**
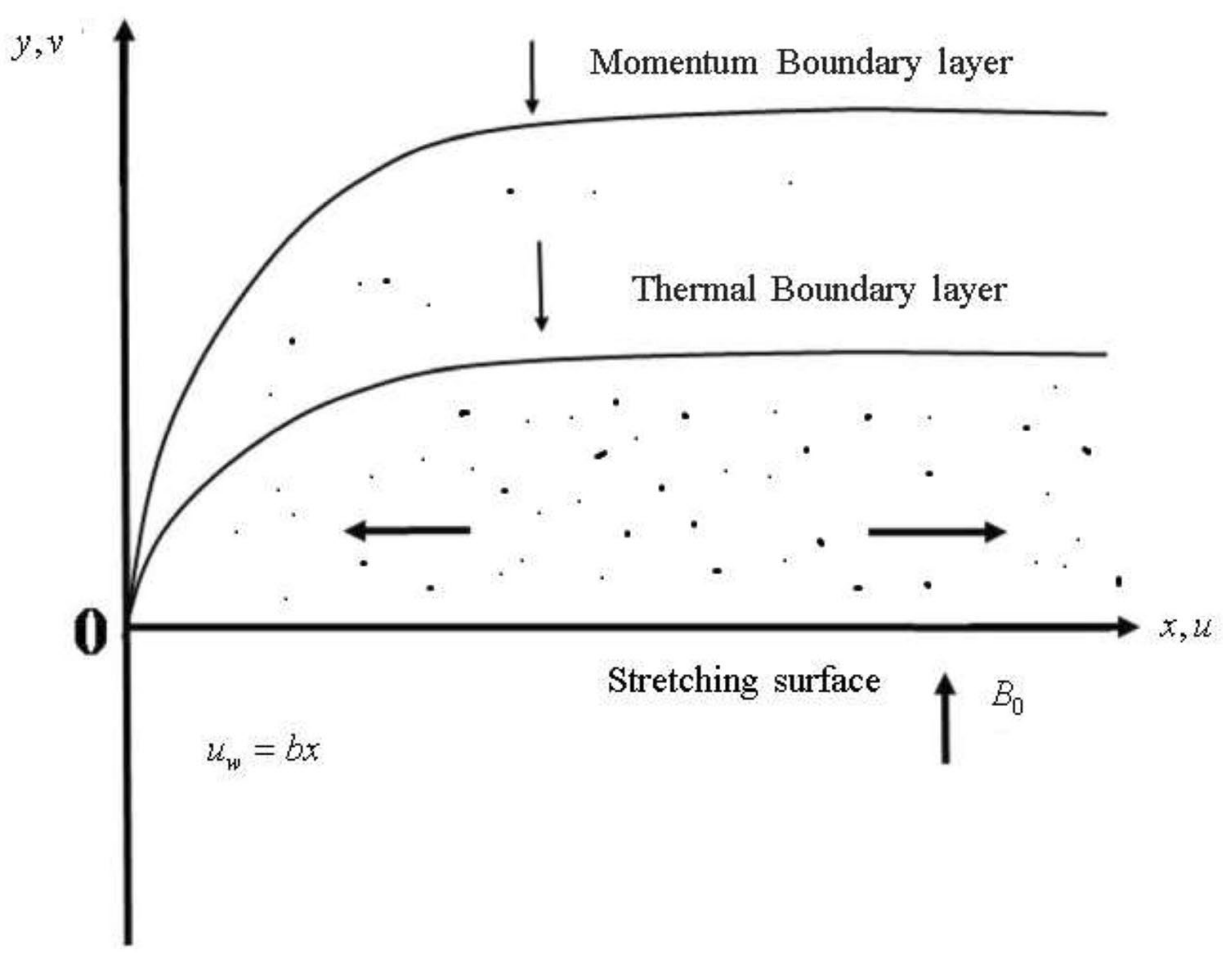
Flow illustration.

The dusty Micropolar, incompressible, steady, MHD nanofluid flow is assumed over an extending sheet with restriction y > 0, and we have considered two forces acting along y- and *x-*direction respectively. where the *y-*axis is considered to be normal in the flow direction. *B*_*0*_ is the magnetic field. The outline of the proposed mathematical model is given in Fig. 1.

Following Oberbeck-Boussinesq, the boundary layer approximation, and the notion that the dust particles have the same size and their density remains constant throughout the fluid flow, the governing system of equations for the fluid phase and the dust particles is given in the subsequent set of equations:

### The Fluid Phase^1,35^

1 $$u_{x} + v_{y} = 0,$$

2 $$uu_{x} + vu_{y} = \nu u_{yy} + \frac{{k^{*} }}{\rho }E_{y} - \frac{{\sigma B_{0}^{2} }}{\rho }u + \frac{{\rho_{p} }}{{\rho \tau_{m} }}\left( {u_{p} - u} \right),$$

3 $$uE_{x} + vE_{y} = \frac{\varepsilon }{{\rho_{j} }}E_{yy} - \frac{{k^{*} }}{{\rho_{j} }}\left( {2E + u_{y} } \right),$$

4 $$\begin{aligned} & uT_{x} + vT_{y} + \lambda_{1} \left[ {u^{2} T_{xx} + v^{2} T_{yy} + 2uvT_{xy} + \left( {uu_{x} + vu_{y} } \right)T_{x} + \left( {uv_{x} + vv_{y} } \right)T_{y} } \right] \\ & \quad = \alpha T_{yy} + \frac{{\rho_{p} c_{s} }}{{\tau_{T} }}\left( {T_{p} - T} \right) + \frac{{Q_{0} }}{{\rho c_{p} }}\left( {T - T_{\infty } } \right) + \tau D_{B} C_{y} T_{y} + \frac{{\tau D_{T} }}{{T_{\infty } }}T_{y}^{2} , \\ \end{aligned}$$

5 $$uC_{x} + vC_{y} = D_{B} C_{yy} + \frac{{D_{T} }}{{T_{\infty } }}T_{yy} - K_{r} \left( {C - C{}_{\infty }} \right) + \frac{{m\rho_{p} }}{{\rho \tau_{c} }}\left( {C_{p} - C)} \right).$$

### The dust phase

6 $$u_{px} + v_{py} = 0,$$

7 $$u_{p} u_{{p_{x} }} + v_{p} u_{{p_{y} }} = - \frac{1}{{\tau_{m} }}\left( {u_{p} - u} \right),$$

8 $$\rho_{p} c_{s} \left( {u_{p} T_{{p_{x} }} + v_{p} T_{{p_{y} }} } \right) = \frac{{ - \rho_{p} c_{s} }}{{\tau_{T} }}\left( {T_{p} - T} \right),$$

9 $$u_{p} C_{px} + v_{p} C_{py} = \frac{{ - m\rho_{p} }}{{\rho \tau_{c} }}\left( {C_{p} - C} \right).$$

The correlated boundary conditions are presented as below:

10 $$\begin{aligned} & u = bx + \alpha^{*} u_{y} ,\,\,v = 0,\,\,E = - n_{1} u_{y} ,\,\,\, - kT_{y} = h_{f} (T_{f} - T),\,\,D_{B} C_{y} + \frac{{D_{T} }}{{T_{\infty } }}T_{y} = 0,\,\,\,\,{\text{at}}\,y\, = \,0 \\ & u \to 0,\,\,u_{p} \to 0,\,\,\,v_{p} \to v,\,\,E \to 0,\,\,T \to T_{\infty } ,\,\,C \to C_{\infty } ,\,\,T_{p} \to T_{\infty } ,\,\,C_{p} \to C_{\infty } .\,\,\,\,{\text{as}}\,\,\,y \to \infty \\ \end{aligned}$$

The non-dimensional form of the above-stated system phases may be obtained by introducing the subsequent transformations:

11 $$\begin{aligned} u & = bxF^{\prime},\,\,\,\,u_{p} = bxF^{\prime}_{p} ,\,\,\,\,v_{p} = - \,\sqrt {\nu b} F_{p} ,\,\,\,\,\,\,v = - \,\sqrt {\nu b} F,\,\,\,\,\,E = bx\sqrt {\frac{b}{\nu }} g(\eta ),\,\,\, \\ \theta_{p} & = \frac{{T_{p} - T_{\infty } }}{{T_{f} - T_{\infty } }},\,\,\,\,\,\theta = \frac{{T - T_{\infty } }}{{T_{f} - T_{\infty } }}\,\,\,\,\phi = \frac{{C - C_{\infty } }}{{C_{w} - C_{\infty } }},\,\,\,\,\phi_{p} = \frac{{C_{p} - C_{\infty } }}{{C_{w} - C_{\infty } }}\,\,\,\,\,\,\eta = \sqrt {\frac{b}{\nu }} y \\ \end{aligned}$$

Using Eq. (11) Eqs. (2)–(10) become:

12 $$F^{\prime\prime\prime} + FF^{\prime} - F^{{\prime}{2}} - MF^{\prime} + Bg^{\prime} + D_{p} \alpha_{d} \left( {F_{p} ^{\prime} - F^{\prime}} \right) = 0$$

13 $$\lambda g^{\prime\prime} - \frac{\lambda }{{G^{*} }}\left( {2g + F^{\prime\prime}} \right) + Fg^{\prime} - f^{\prime}g = 0$$

14 $$\theta ^{\prime\prime} - \Pr \gamma \left( {FF^{\prime}\theta ^{\prime} + F^{2} \theta ^{\prime\prime}} \right) + \Pr F\theta ^{\prime}M + D_{p} \alpha_{d} \left( {\theta_{p} - \theta } \right) + Q\theta + N_{b} \theta ^{\prime}\phi ^{\prime} + N_{t} \theta ^{{\prime}{2}} = 0,$$

15 $$\phi ^{\prime\prime} + ScF\phi ^{\prime} + \frac{{N_{t} }}{{N_{b} }}\theta ^{\prime\prime} - K_{c} \phi + Sc\beta_{c} l\left( {\phi_{p} - \phi } \right) = 0,$$

16 $$F_{p} F_{p} ^{\prime\prime} + \alpha_{d} \left( {f^{\prime} - F_{p} ^{\prime}} \right) = 0.$$

17 $$F_{p} \theta_{p} ^{\prime} + \frac{1}{\Gamma \Pr }\alpha_{d} \left( {\theta - \theta_{p} } \right) = 0.$$

18 $$F_{p} \phi_{p}^{\prime } + \beta_{c} l\left( {\phi_{p} - \phi } \right) = 0$$

19 $$\begin{aligned} F^{\prime}(0) & = 1 + \delta F^{\prime\prime},\,\,\,\,\,g(0) = - n_{1} F^{\prime\prime},\,\,\,\theta ^{\prime}(0) = - B_{1} \left( {1 - \theta (0)} \right),\,\,\,N_{b} \phi ^{\prime}(0) + N_{t} \theta ^{\prime}(0) = 0, \\ F^{\prime}(\infty ) & = 1,\,\,\,F_{p} = F,\,\,\,\,\,\,\,\theta (\infty ) = 0,\,\,\,\theta_{p} (\infty ) = 0,\,\,\,\,\,\,\phi (\infty ) = 0,\,\,\,\,\,\,\phi_{p} (\infty ) = 0. \\ \end{aligned}$$

The quantities defined above are given by:

20 $$\begin{aligned} \Pr & = \frac{\upsilon }{\alpha },\,\,G^{*} = \frac{\varepsilon b}{{k^{*} \nu }},\,\,\,\,\lambda = \frac{\varepsilon }{{\rho_{j} \nu }},\,\,\,\,\gamma = \lambda_{1} b,M = \frac{{\sigma B_{0}^{2} }}{\rho b},D_{p} = \frac{{\rho_{p} }}{\rho },\,\,\,\,\,\alpha_{d} = \frac{1}{{\tau_{m} b}}, \\ N_{b} & = \frac{{\tau D_{B} C_{\infty } }}{\nu },\,\,N_{t} = \frac{{\tau D_{T} \Delta T}}{{T_{\infty } \nu }},\,\,Sc = \frac{\upsilon }{{D_{B} }},\,\,\,\Gamma = \frac{{c_{s} }}{{c_{p} }},\,\,\,\delta = \alpha *\sqrt {\frac{b}{\nu }} ,\,\,\,\beta_{c} = \frac{1}{{\tau_{c} }}, \\ l & = \frac{{m\rho_{p} }}{\rho },\,\,\,B_{1} = \frac{{h_{s} }}{k}\sqrt {\frac{b}{\nu }} ,\,\,\,\,\,Q = \frac{{Q_{0} }}{{\rho c_{p} b}},K_{c} = \frac{{K_{r} }}{b}. \\ \end{aligned}$$

Drag force coefficient in ($C_{F}$) and Sherwood number ($Sh_{x}$),are given by:

21 $$C_{F} = \frac{{\tau_{w} }}{{\rho u_{{_{\infty } }}^{2} }},\,\,\,Sh_{x} = \left. {\frac{{xq_{n} }}{{D_{n} (C_{w} - C_{\infty } )}}} \right|_{y = 0} .$$

where

22 $$\tau_{w} = \mu \left. {u_{y} } \right|_{y = 0} , \quad q_{n} = \left. { - D_{B} C_{y} } \right|_{y = 0} ,$$

The dimensionless forms of the aforementioned physical quantities are stated as under:

23 $$\sqrt {{\text{Re}}_{x} } C_{F} = F^{\prime\prime}(0)\,\,Sh\sqrt {{\text{Re}}_{x} } = - \phi ^{\prime}(0).$$

## Numerical solution

The numerical methodology of MATLAB software bvp4c is implemented to evaluate the transformed coupled non-linear ordinary differential equations. The method bvp4c method possesses the following characteristics:

1. It is simple to use and has a quick convergence rate.
2. It has a reduced computing cost and, in comparison to other analytical techniques, a higher degree of accuracy.
3. For some problems, the shooting technique is unhelpful because it is sometimes very sensitive to early guesses and bvp4c, on the other hand, uses a collocation method that is more reliable than shooting.

**Figure 2:**
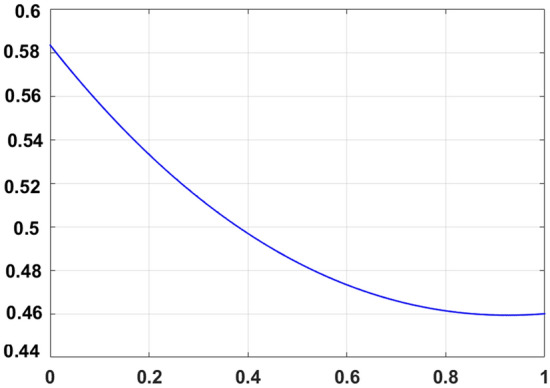
Mesh model.

With a mesh size, $h = 0.1,$ the bvp4c method is used for ameliorate approximations (Fig. 2). The technique is authentic if the auxiliary conditions are fulfilled with a precision of $10^{ - 6}$.

First of all, new variables are introduced as:

24 $$\begin{aligned} y_{1} & = F,y_{2} = F^{\prime},y_{3} = F^{\prime\prime},yy_{1} = F^{\prime\prime\prime},y_{4} = F_{p} ,y_{5} = F^{\prime}_{p} ,yy_{2} = F^{\prime\prime}_{p} ,y_{6} = g,y_{7} = g^{\prime},yy_{3} = g^{\prime\prime},y_{8} = \theta , \\ y_{9} & = \theta^{\prime},yy_{4} = \theta^{\prime\prime},y_{10} = \theta_{p} ,yy_{5} = \theta_{p} ,y_{11} = \phi ,y_{12} = \phi^{\prime},yy_{6} = \phi^{\prime\prime},y_{13} = \phi_{p} ,yy_{7} = \phi_{p}^{\prime } , \\ \end{aligned}$$

The following equations can be obtained using the above equations in the MATLAB bvp4c technique:

25 $$yy_{1} = - y_{1} y_{2} + y_{2}^{2} + My_{2} - By_{7} - D_{p} \alpha_{d} \left( {y_{5} - y_{2} } \right),$$

26 $$yy_{2} = \frac{{ - \alpha_{d} \left( {y_{2} - y_{5} } \right)}}{{y_{4} }},$$

27 $$yy_{3} = \left( {\frac{{\frac{\lambda }{G*}\left( {2y_{6} + y_{3} } \right) - y_{2} y_{7} + y_{2} y_{6} }}{\lambda }} \right),$$

28 $$yy_{4} = \left( {\frac{{\left( {\Pr \gamma \left( {y_{1} y_{2} y_{9} } \right)} \right) - \Pr My_{1} y_{9} + y_{2}^{2} - D_{p} \alpha_{d} \left( {y_{10} - y_{8} } \right), - Qy_{8} - N_{b} y_{9} y_{12} - N_{t} y_{9}^{2} }}{{1 + \Pr \gamma y_{1}^{2} }}} \right),$$

29 $$yy_{5} = \frac{{\left( {\frac{ - 1}{{\Gamma \Pr }}\alpha_{d} \left( {y_{8} - y_{10} } \right)} \right)}}{{y_{4} }},$$

30 $$yy_{6} = - Scy_{1} y_{12} - \frac{{N_{t} }}{{N_{b} }}yy_{4} + K_{c} y_{11} + Scl\beta_{c} \left( {y_{13} - y_{11} } \right),$$

31 $$yy_{7} = \frac{{l\beta_{c} \left( {y_{13} - y_{11} } \right)}}{{y_{4} }},$$

with the transmuted boundary conditions

32 $$\begin{aligned} & y_{1} (0) - 1 - \delta y_{3} (0),y_{6} (0) + ny_{3} (0),y_{9} (0) + B_{1} \left( {1 - y_{8} (0)} \right),N_{b} y_{12} (0) + N_{t} y_{9} (0), \\ & y_{2} \left( \infty \right) - 1,y_{4} \left( \infty \right) - y_{1} \left( \infty \right),y_{8} \left( \infty \right),y_{10} \left( \infty \right),y_{11} \left( \infty \right),y_{13} \left( \infty \right), \\ \end{aligned}$$

**Figure 3:**
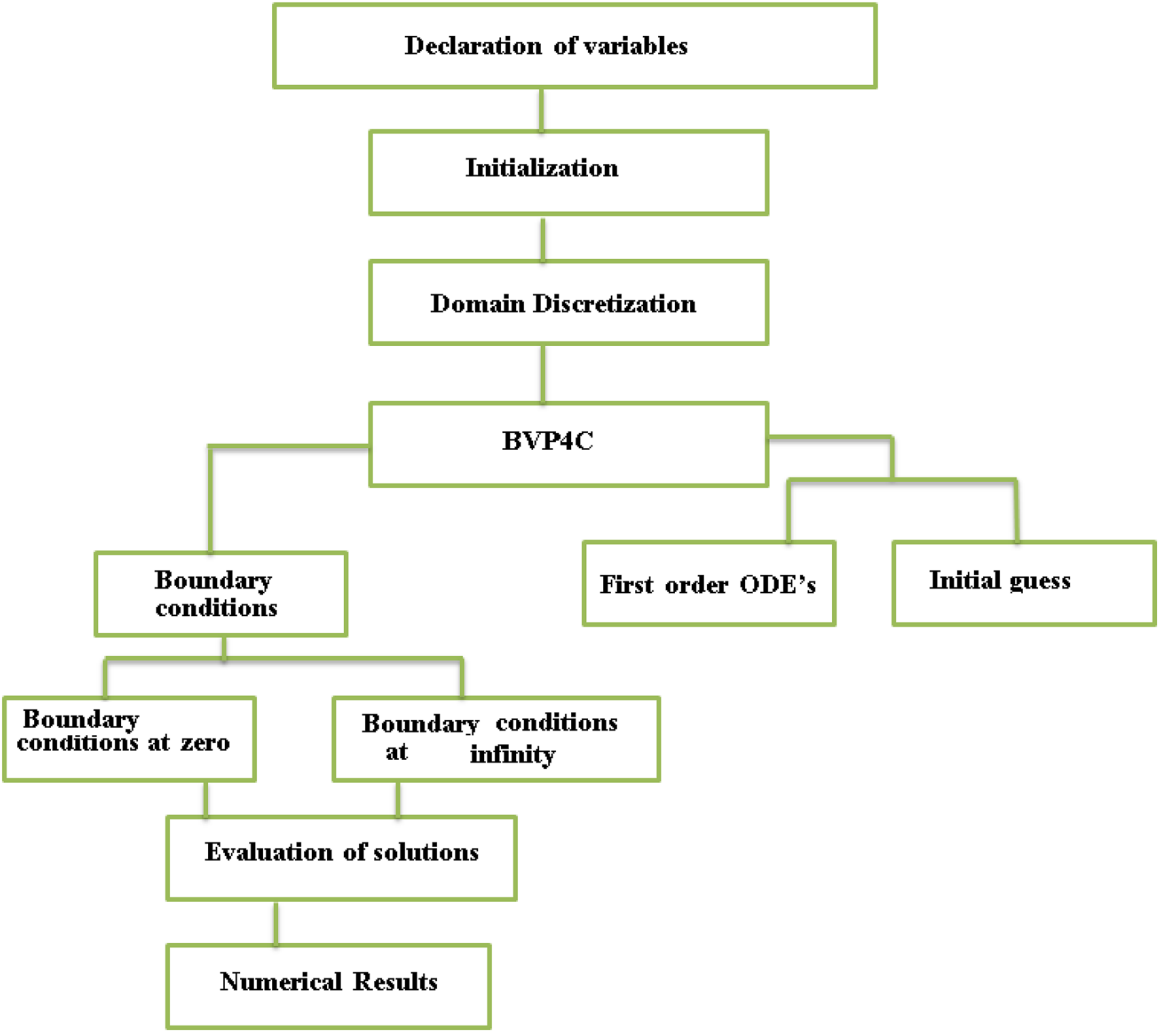
Flow chart of the numerical scheme.

The flow chart (Fig. 3) of the implemented numerical scheme is appended as under:

## Outcomes with discussion

**Figure 4:**
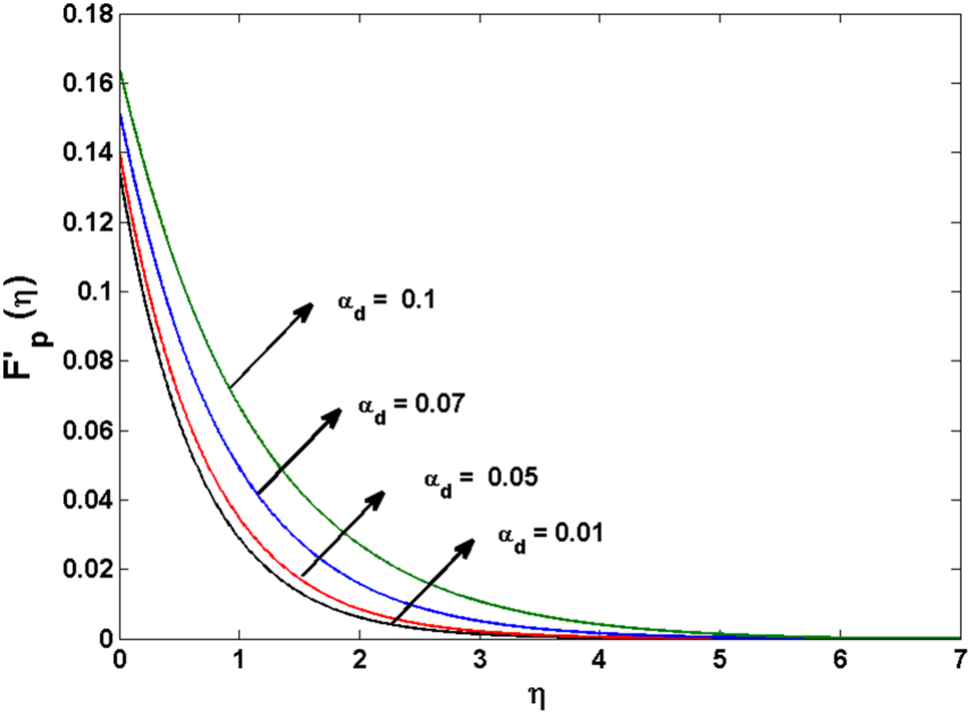
$F^{\prime}_{p} \left( \zeta \right)$ for various estimates of $\alpha_{d}$.

**Figure 5:**
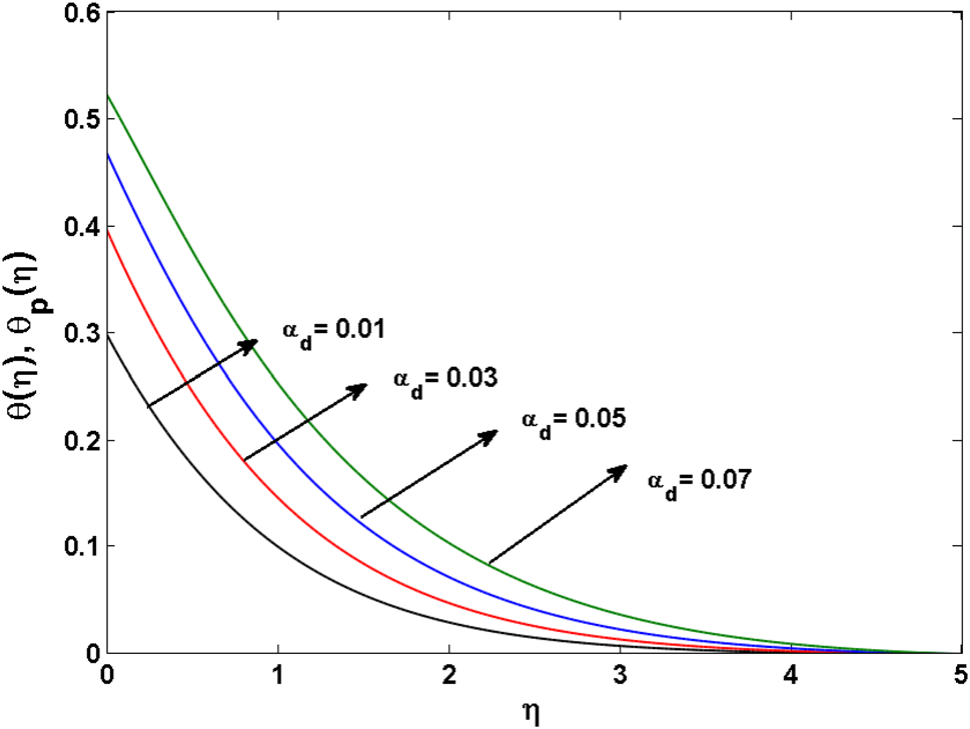
$\theta^{\prime}_{p} \left( \zeta \right)$ for various estimates of $\alpha_{d}$.

**Figure 6:**
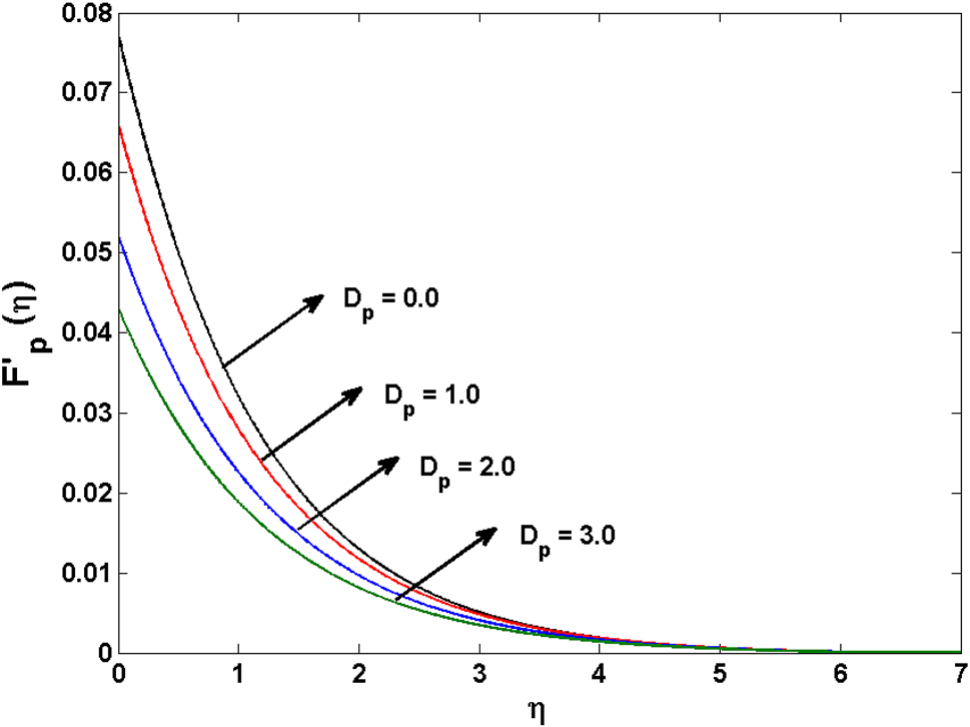
$F^{\prime}_{p} \left( \zeta \right)$ for various estimates of $D_{p}$.

**Figure 7:**
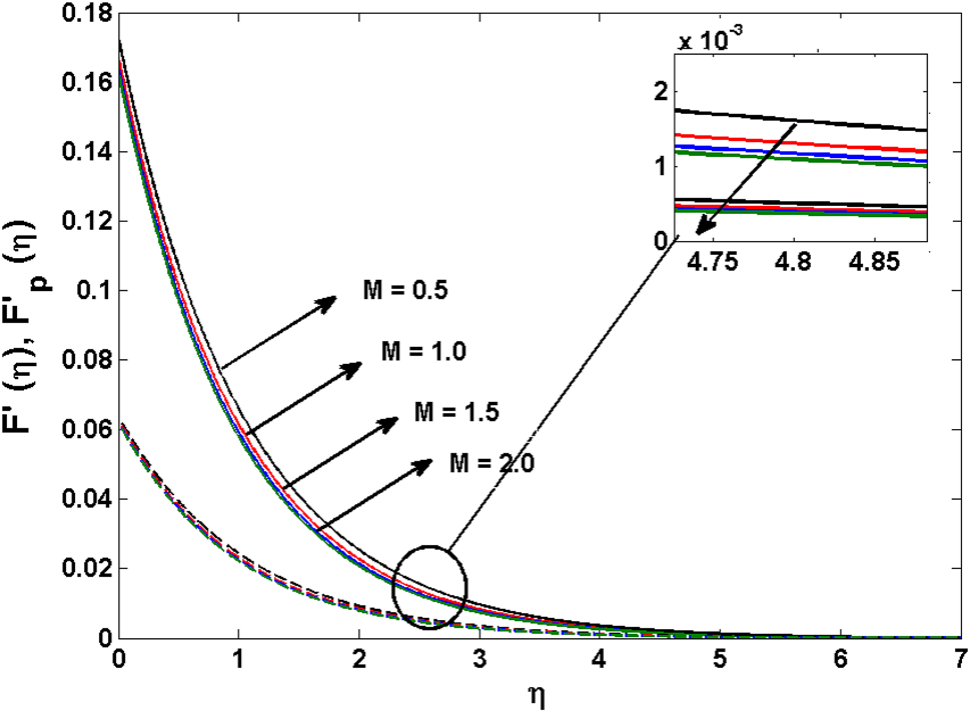
$F^{\prime}\left( \eta \right)$ and $F^{\prime}_{p} (\eta )$ for various estimates of $M$.

**Figure 8:**
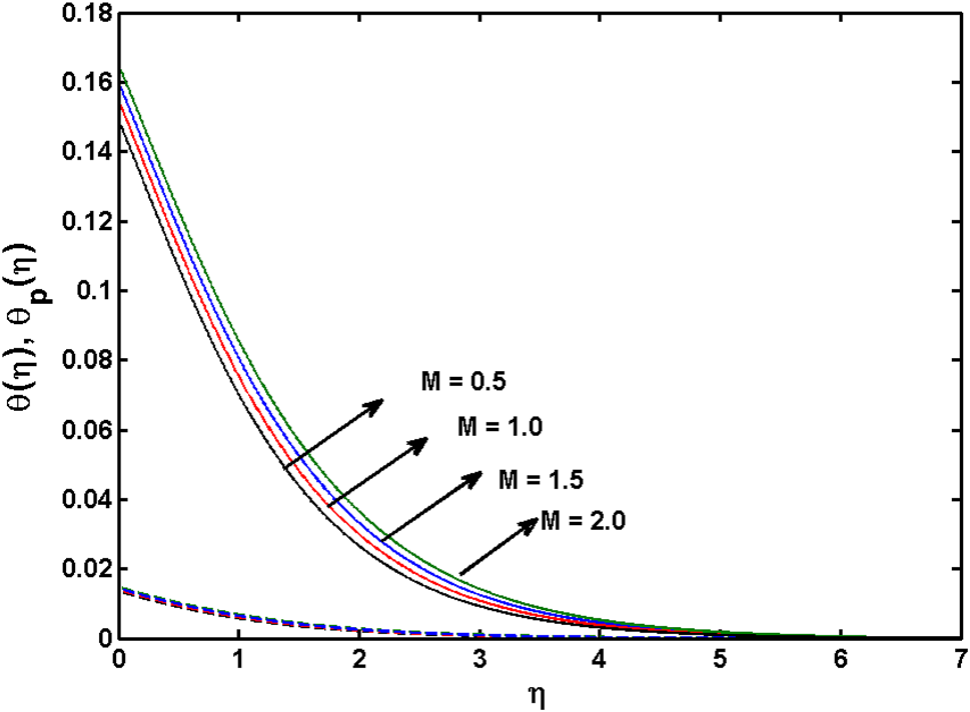
$\theta \left( \eta \right)$ and $\theta_{p} (\eta )$ for various estimates of $M$.

**Figure 9:**
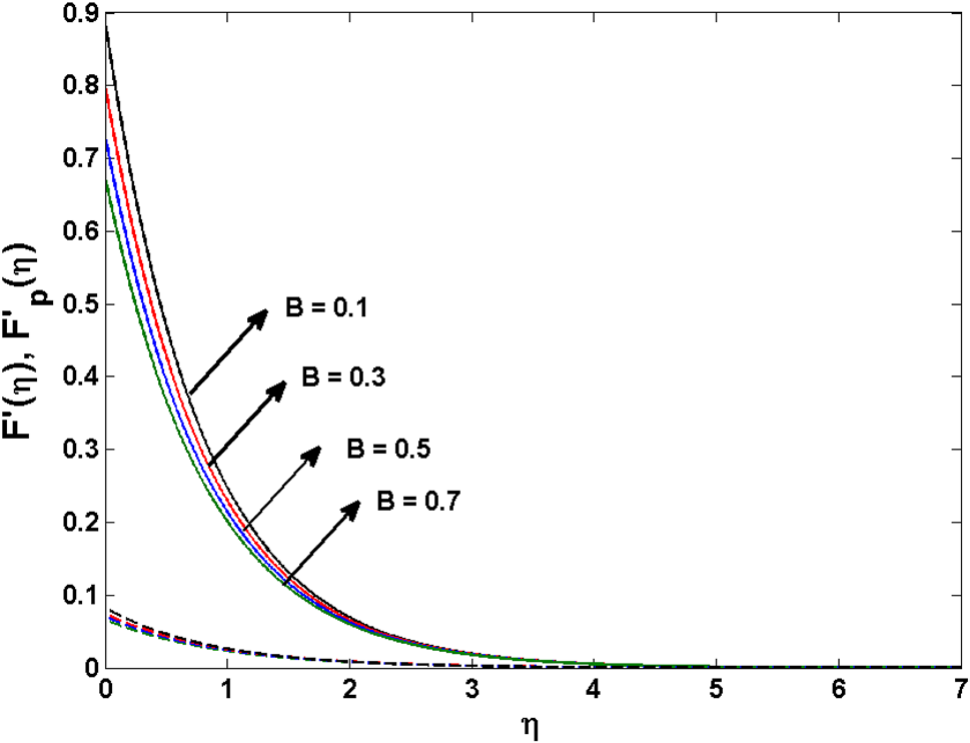
$F^{\prime}\left( \eta \right)$ and $F^{\prime}_{p} (\eta )$ for various $B$.

**Figure 10:**
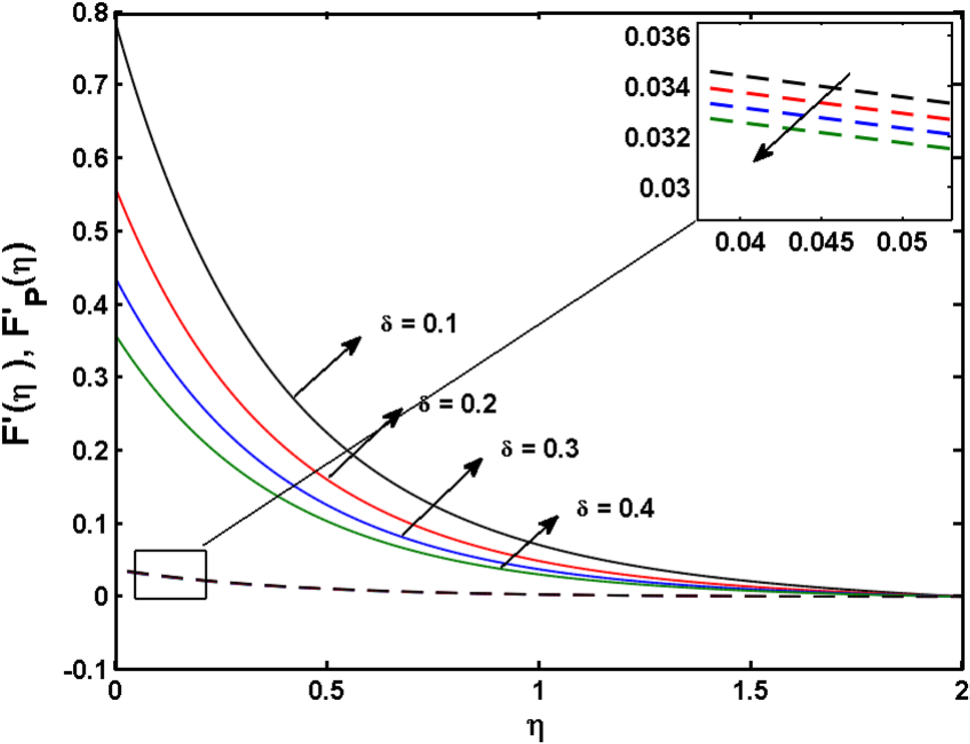
$F^{\prime}\left( \eta \right)$ and $F^{\prime}_{p} (\eta )$ for various estimates of $\delta$.

**Figure 11:**
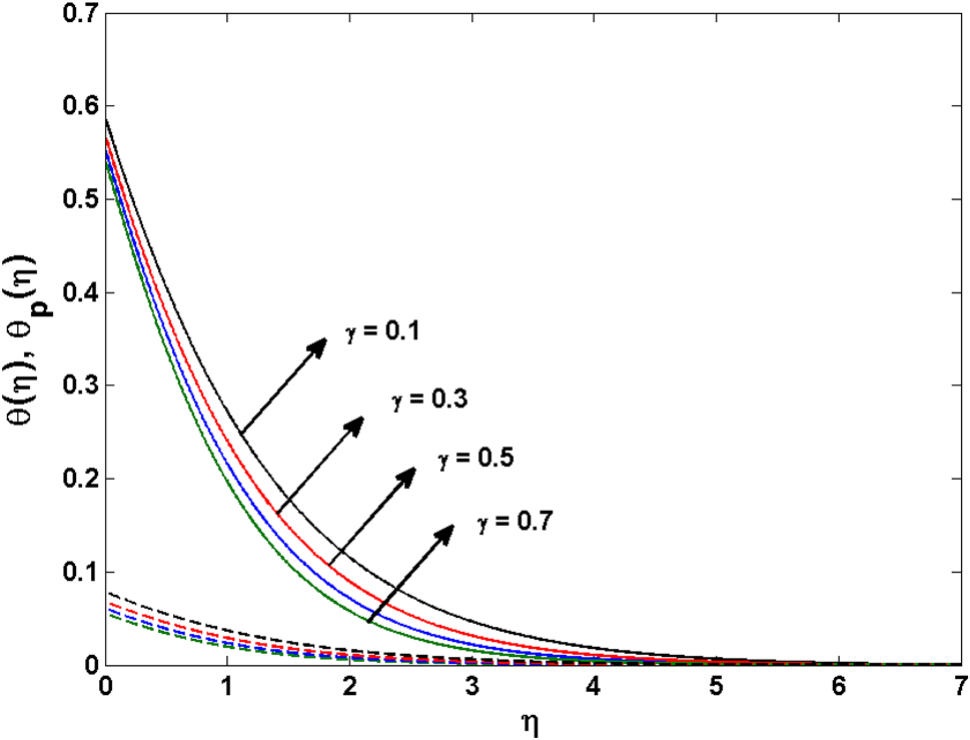
$\theta \left( \eta \right)$ and $\theta_{p} (\eta )$ for various estimates of $\gamma$.

**Figure 12:**
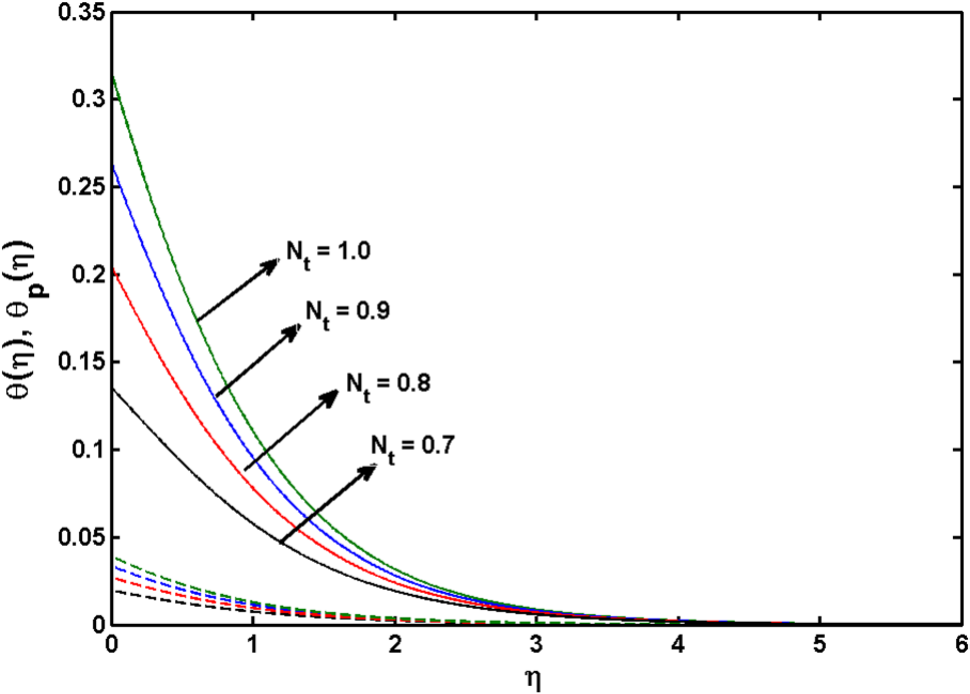
$\theta \left( \eta \right)$ and $\theta_{p} (\eta )$ for various estimates of $N_{t}$.

**Figure 13:**
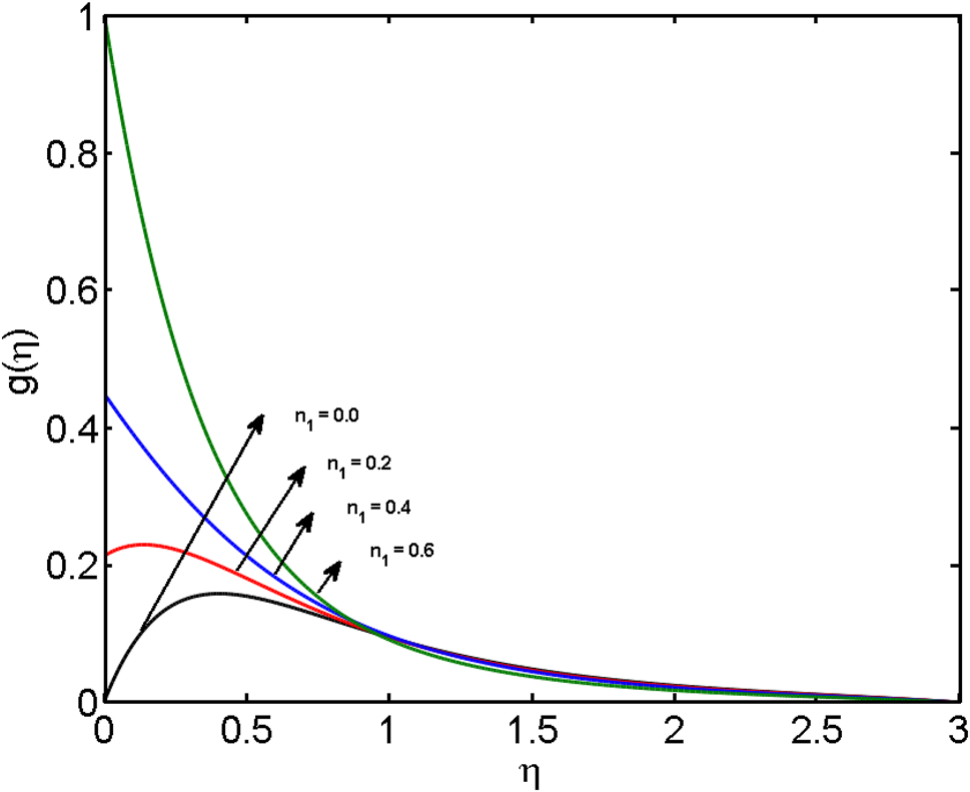
$g(\eta )$ for various estimates of $n_{1}$.

**Figure 14:**
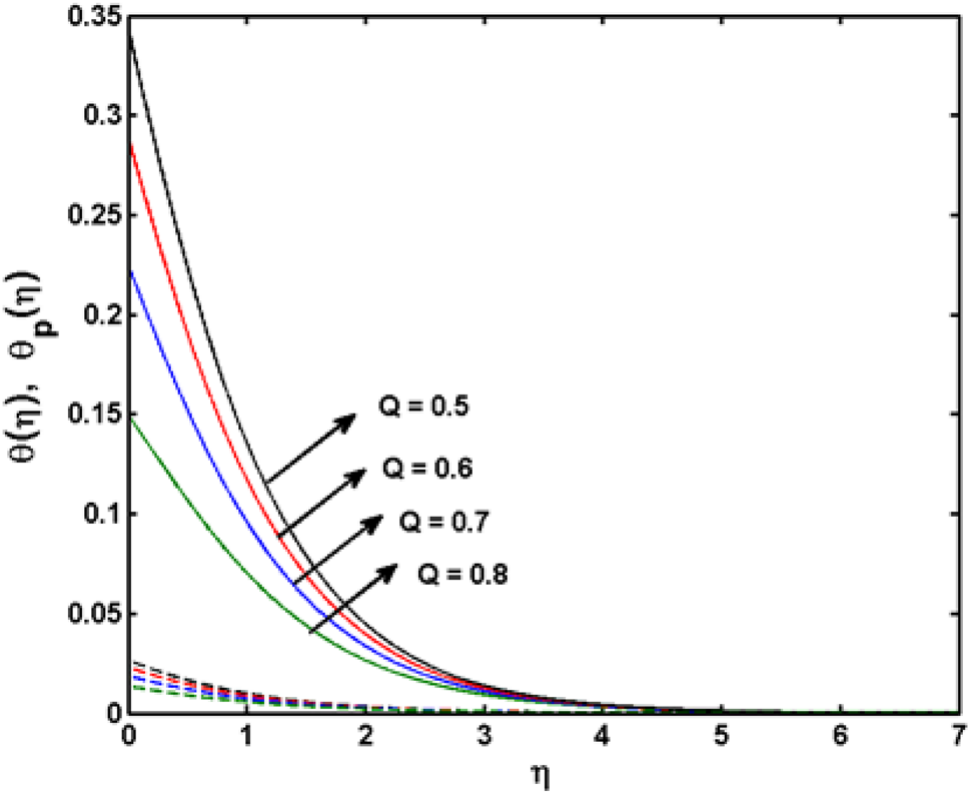
$\theta \left( \eta \right)$ and $\theta_{p} (\eta )$ for various estimates of $Q$.

**Figure 15:**
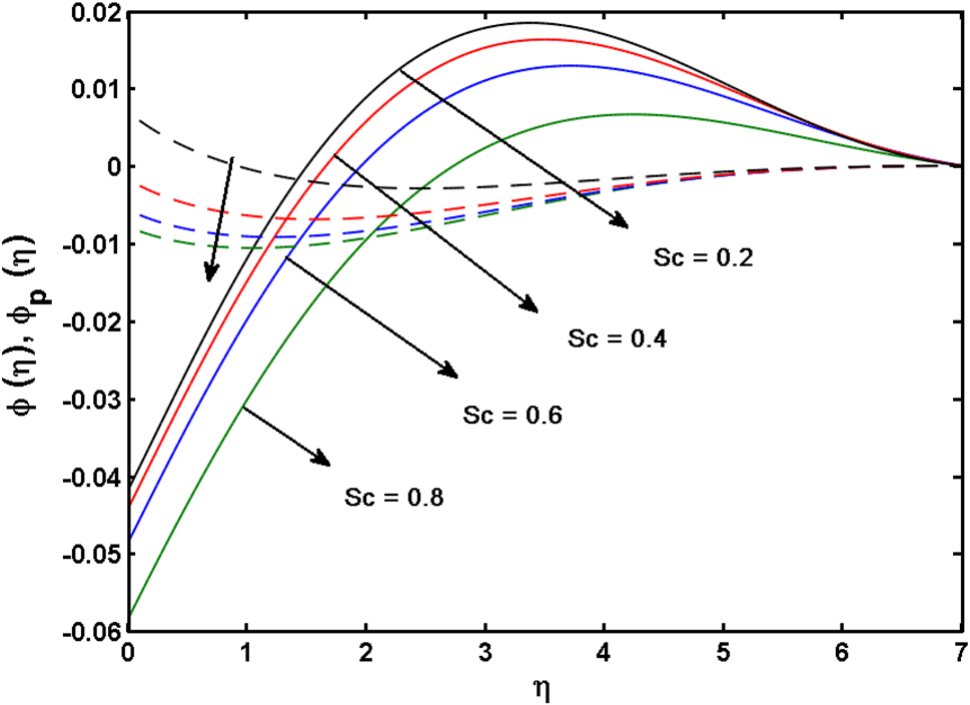
$\phi (\eta )$ for various estimates of $Sc$.

**Figure 16:**
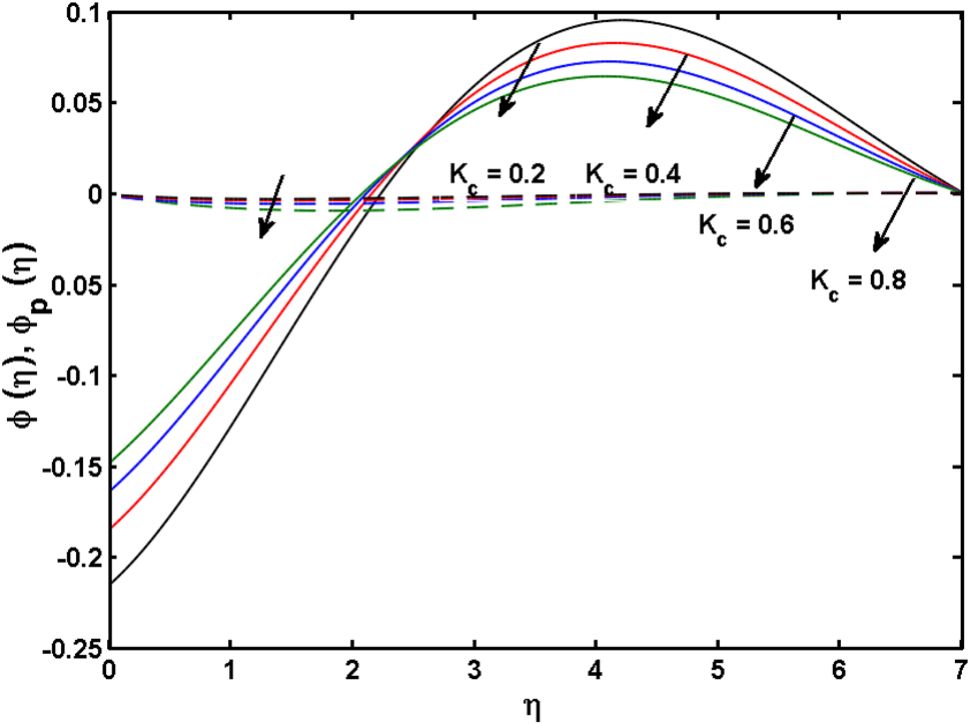
$\phi (\eta )$ for various estimates of $K_{c}$.

**Figure 17:**
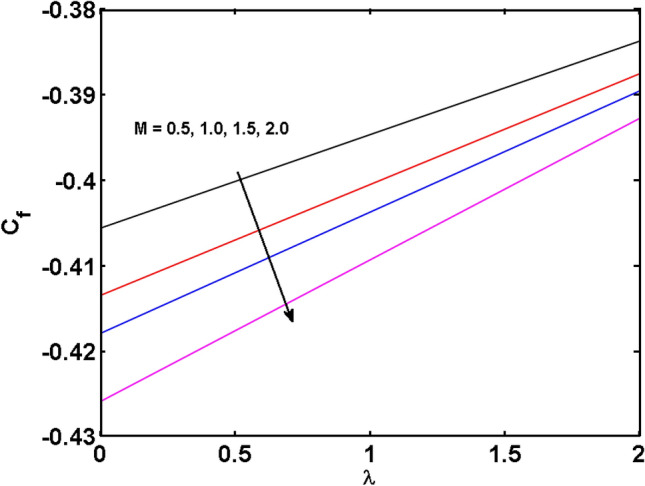
$C_{F}$ for various of estimates of $M$ and $\lambda$.

**Figure 18:**
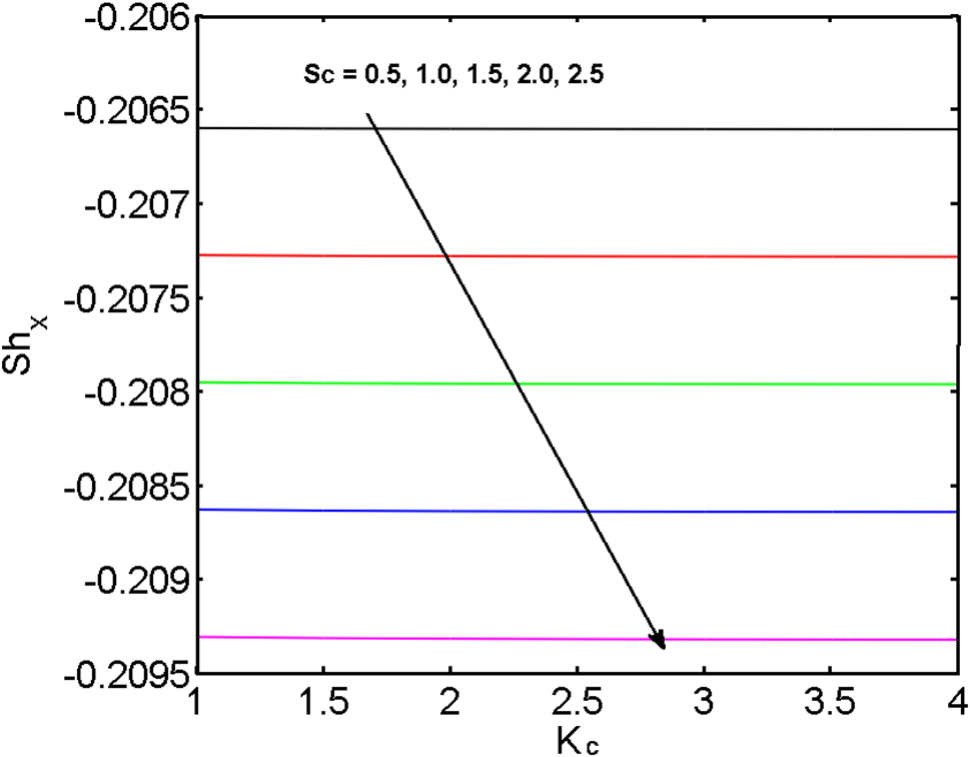
$Sh_{x}$ for various estimates of $K_{c}$ and $Sc$.

This segment (Figs. 4, 5, 6, 7, 8, 9, 10, 11, 12, 13, 14, 15, 16, 17, 18) is developed to assess the evident attributes of the leading emergent parameters on the related profiles. Figures 4 and 5 depict that the velocity and temperature of dust particles surge as the fluid-particle interaction parameter $\alpha_{d}$ upsurges. This behavior can be caused by the fact that when the interaction between fluid and particles is high and the particle phase has thermal conductivity hegemony, the particle phase declines the liquid velocity till it reaches the same liquid velocity. This results in a fall in the fluid velocity and an upsurge in the velocity of dust particles. Figure 6 illustrates the influence of dust particle mass concentration ($D_{p}$) in the velocity field for the dust phase. It is observed that by growing $D_{p}$, the skin friction rises which causes difficulty in the movement of the nanofluid. Thus, $F^{\prime}_{p} \left( \eta \right)$ declines. Figures 7 and 8 show the dimensionless velocity and thermal profiles in both dust and liquid phases for various estimations of the magnetic parameter (*M*). With increasing estimations of *M*, it is clear that the dimensionless velocities ($F^{\prime}$ and $F^{\prime}_{p}$) shrink, while the dimensionless temperatures ($\theta$ and $\theta_{P}$) expand respectively. Physically, substantial Lorentz force initiates resistance in the liquid motion and the fluid develops more viscous that’s why the velocity profile lowers. It is noted that for increasing values of *M,* the magnetic field has a thickening outcome on the thermal boundary layer which gives escalation to temperature. Figure 9 shows the velocity profile in both dust and liquid phases for distinct values of the coupling constant parameter (*B*). It is evident from this graph that both velocities (dust and liquid) are declining under the influence of *B*. The effect of the velocity slip parameter $(\delta )$ on the velocity profile is demonstrated in Fig. 10. The boundary layer thickness and velocity are found to drop as the $\delta$ is increased. When $\delta$ increases, some of the stretching velocity is shifted to the liquid. As a consequence, the velocity profile reduces. The sway of the thermal convection parameter $(\gamma )$ on the fluid temperature is exhibited in Fig. 11. It is illustrated that both liquid and dust phases thickness of the thermal boundary layer are lessened for mounting estimates of $\gamma$. Greater values of relaxation times result in non-conductive behavior of the material which is liable for decay in the thermal profile. Figure 12 indicates the effect of the thermophoresis parameter $(N_{t} )$ on the thermal profile. The higher temperature is seen for large estimations of $N_{t}$. This is due to an increase in the number of nanoparticles of fluid approaching the hot surface, causing the temperature profile to rise. In Fig. 13, the estimation of angular velocity $(n_{1} )$ increases for higher values of *n*. For $n_{1} = 0$ leads to $g = 0$ which indicated that there is no-spin condition according to the boundary condition at the wall, $g(0) = - n_{1} F^{\prime\prime},\,$ this means that the microelements in the concentrated particle flow near the wall surface are unable to rotate. For $n_{1} = 0.5$ when $g \ne 0,$ it indicates that the anti-symmetric component of the stress tensor disappears and is replaced by a weak concentration. The particle spin must be comparable to the fluid velocity at the wall in fine particle movements. The impact of sink/source parameter (*Q*) versus thermal profile is displayed in Fig. 14. It is illustrated that the thermal profile reduces for greater estimation of *Q*. Figure 15 is outlined to study the impact of Schmidt number $(Sc)$ on the concentration profile. For greater valuations of $Sc$ feeble concentration is noticed. Greater values of $Sc$ result in smaller Brownian diffusivity. This weak Brownian diffusivity will lower the concentration field. In Fig. 16, the impact of chemical reaction $K_{c}$ on concentration field is addressed. Here, one can observe that the concentration field decreases for large estimations of $K_{c}$ . It is perceived that greater values of $K_{c}$ result in decay in the concentration profile. Figure 17 illustrated the influence on the surface Drag force coefficient $C_{F}$ for $M$ and $\lambda$. It is noticed that the $C_{F}$ decreases versus growing values of $M.$ The influence of $Sh_{x}$ for $K_{c}$ and $Sc$ is revealed in Fig. 18. It is examined that $Sh_{x}$ decline for greater $Sc$. Table 2 depict the comparison of magnetic parameter M with Akbar et al.^34^ and Gireesha et al.^35^. The outcomes are found in an outstanding agreement.

**Table 2:** Comparison values of Skin friction co-efficient (*B* = 0).

M	^15^	^16^	Present
01	− 1.41421	− 1.41421	− 1.41420
05	− 2.44948	− 2.44949	− 2.44949
10	− 3.31662	− 3.31662	− 3.31664
50	− 7.14142	− 7.14143	− 7.14140
500	− 22.3830	− 22.38302	− 22.3831
1000	− 31.6386	− 31.63858	− 31.6359

## Final remarks

In this research, we have examined the role of modified Fourier law in the flow of an MHD Micropolar nanoliquid flow with dust particles over a stretched surface. The distinctiveness of the presented model is boosted with additional impacts of the chemical reaction and the heat source/sink with slip, convective and zero mass flux conditions at the boundary. The erected model is handled numerically with the bvp4c function of MATLAB software. The results are obtained graphically for the associated profiles versus respective parameters and discussed logically. A comparison is also made with a published paper to ascertain the validity of the presented model. The answers to the above raised questions with other salient highlights are appended as:

- The velocity and temperature of dust particles rise as the fluid-particle interaction parameter increases.
- For positive values of magnetic parameter, dimensionless velocity decreases while temperature profile increases in both dust and liquid phases.
- In both dust and liquid phases, the thermal boundary layer thickness is lessened for growing estimates of thermal relaxation time.
- The thickness of the boundary layer and velocity was found to decline as the velocity slip parameter is heightened.
- The mass transfer rate reduces by escalating the Schmidt number and chemical reaction parameter.
- The fluid and dust phases are enhanced for gradual escalated estimations of the heat source/sink parameter.

## List of symbols

$B_{0}$: Magnetic field strength
$$k*$$: Rotational coefficient
$\rho_{j}$: Density of micro-inertia
$\tau_{m}$: Velocity relaxation time
$C$: Concentration of the fluid
$C_{w}$: Concentration of nanoparticles
$C_{\infty }$: Free stream concentration of nanoparticles
$$N_{t}$$: Thermophoresis parameter
$$\Pr$$: Prandtl number
$$c_{p}$$: Specific heat
$c_{s}$: Specific heat of dust particles
$$K_{r}$$: Chemical reaction
$$x,y$$: Coordinate axis
$D_{B}$: Brownian diffusion coefficient
$$E$$: Angular velocity
$Re_{x}$: Local Reynold number
$T$: Temperature of fluid
$$q_{n}$$: Mass flux
$\tau_{c}$: Mass relaxation of a dust particle
$h_{f}$: Heat transfer coefficient
$$B_{1}$$: Biot number
$T_{p}$: Dust particles temperature
$\rho_{p}$: Density of dust particle
$C_{p}$: Dust particles concentration
$$\alpha *$$: Slip coefficients
$$M$$: Magnetic parameter
$D_{p}$: Ration of density of nanofluid to the density of dust particles
$G*$: Micropolar material parameter
$$\tau_{w}$$: Shear stress
$$Sh_{x}$$: Sherwood number
$$\delta$$: Slip parameter
$$k$$: Thermal conductivity
$\varepsilon$: Viscosity of spin gradient
$\rho_{p}$: Density of dust particles
$\sigma$: Electrical conductivity of liquid
$$q_{n}$$: Mass flux
$$l$$: Characteristic length
$m$: Mass of dust particles
$\lambda_{1}$: Thermal relaxation time coefficient
$N_{b}$: Brownian motion parameter
$$T_{\infty }$$: Ambient temperature
$u,v$: Components of velocity
$u_{p} ,v_{p}$: Velocity of dust particles
$Q$: Source/sink parameter
$$\eta$$: Similarity variable
$D_{T}$: Thermophoresis diffusion coefficient
$$\nu$$: Kinematic viscosity
$\rho$: Density of fluid
$\tau$: Ratio of nanoparticles
$T_{p}$: Temperature of a dust particle
$\tau_{T}$: Relaxation time of the dust particle
$\Gamma$: Specific heat ratio of the mixture
$$u_{e}$$: Stretching velocity
$\tau$: Ratio of specific heat
$$b$$: Constant
$$\mu$$: Dynamic viscosity
$B$: Coupling constant parameter
$\lambda$: Ratio of viscosity of spin gradient to the density of particle phase
$\gamma$: Thermal relaxation parameter
$$C_{F}$$: Skin friction
$\alpha_{d}$: Fluid particle interaction parameter
